# Genomic Variability of Hepatitis B Virus Circulating in Brazilian Western Amazon

**DOI:** 10.3390/v14102100

**Published:** 2022-09-22

**Authors:** Tárcio Peixoto Roca, Livia Melo Villar, Felipe Souza Nogueira Lima, Mariana Pinheiro Alves Vasconcelos, Lourdes Maria Pinheiro Borzacov, Eugênia de Castro e Silva, Bárbara Vieira do Lago, Mayara Torquato Lima da Silva, Luan Felipo Botelho Souza, Juan Miguel Villalobos Salcedo, Alcione de Oliveira dos Santos, Deusilene Souza Vieira

**Affiliations:** 1 Laboratory of Viral Hepatitis, Oswaldo Cruz Institute, FIOCRUZ, Rio de Janeiro 21040-900, Brazil; 2 Laboratory of Molecular Virology, Oswaldo Cruz Foundation of Rondônia—FIOCRUZ/RO, Porto Velho 76812-245, Brazil; 3 Tropical Medicine Research Center of Rondônia—CEPEM/RO, Porto Velho 76812-329, Brazil; 4 Laboratory of Biotechnology and Structural Bioengineering, Biophysics Institute Carlos Chagas Filho, Federal University of Rio de Janeiro, Rio de Janeiro 21941-901, Brazil; 5 School of Biomedical Sciences, Aparicio Carvalho University Center, Porto Velho 76811-678, Brazil; 6 Postgraduate Program in Experimental Biology, Federal University of Rondônia—PGBIOEXP/UNIR, Porto Velho 76801-059, Brazil

**Keywords:** mutation, genotype, clinical characterization

## Abstract

The emergence of clinically relevant mutations in the hepatitis B virus (HBV) genome has been a matter of great debate because of the possibility of escape from the host’s immune system, the potential to cause more severe progression of liver diseases and the emergence of treatment-resistant variants. Here we characterized the circulating variants of HBV in Rondônia State, in the north of Brazil. Serum samples of 62 chronic HBV carriers were subjected to PCR assays and clinical data were collected. Mutations and genotypes were characterized through direct sequencing. The findings show the presence of subgenotypes A1 (54.83%, 34/62), D3 (16.13%, 10/62), F2 (16.13%, 10/62), A2 (4.84%, 3/62), D2 (3.23%, 2/62), D1 (1.61%, 1/62), D4 (1.61%, 1/62) and F4 (1.61%, 1/62). Deletions in the pre-S2 region were found in 13.79% (8/58) of the samples, mutations in the S gene in 59.68% (37/62) and RT mutations in 48.39% (30/62). We found a variable genotypic distribution in different locations and important mutations related to immune escape and drug resistance in Western Amazonia, which contributed to genetic surveillance and provided important information to help control the disease.

## 1. Introduction

An estimated two billion people have been exposed to Hepatitis B virus (HBV) worldwide and 257 million individuals are at risk of developing hepatocellular cirrhosis and carcinoma (HCC) from chronic infection [1]. The global strategy of the World Health Organization (WHO) is to eliminate hepatitis B as a public health threat by 2030 [1]. From 1999 to 2019, Brazil registered 247,890 confirmed cases with a detection rate of 6.7 cases per 100,000 inhabitants in 2019 [2]. The state of Rondônia, located in the north is highly endemic [3,4,5] with its capital Porto Velho ranking first among those with the highest detection rates: with 30.4 cases per 100,000 inhabitants, which is higher than the national average [2,6].

HBV belongs to the *Hepadnaviridae* family and has a circular partially double-stranded DNA genome, of about 3.2 kb [7,8]. The viral genome contains four overlapping open reading frames (ORFs) presented as Pre-S/S, Pre-C/C, X, and P, which represent, respectively, the surface, capsid, X and viral polymerase proteins [9,10]. HBV has been classified into 10 genotypes (A–J) based on a whole genome divergence intergroup greater than 8%; and several subgenotypes with a divergence of less than 4% between sub-groups [11,12]. Due to the lack of corrective activity by the viral polymerase, HBV isolates are subjected to an expressive genetic variability. Mutations that influence immune escape, disease outcome, carcinogenesis and treatment resistance occur in specific regions of the genome [13,14,15,16].

HBV infection in adulthood follows an acute course with complete recovery in most cases (90–95%) and rarely a fulminant course (less than 1% of individuals). If the infection persists for more than 6 months, it can become chronic (5–10% of cases) leading to progressive liver fibrosis, cirrhosis and HCC [17,18,19]. The natural course of chronic hepatitis B (CHB) can be classified into phases: Immune-Tolerant, Immune-Active, Inactive, HBV reactivation [18,20]. Clinical manifestations may vary according to viral and host features such as age, sex, immune host background, genetic variability, the amount of virus at the time of infection and the presence of coinfections [21,22].

The genetic variability of HBV and the clinical outcomes of patients in the Western Amazon is still understudied. In this context, this study characterized the circulating genotypes, clinically relevant mutations, and demographic and clinical features in chronic hepatitis B carriers from Rondônia state.

## 2. Materials and Methods

### 2.1. Study Population

The study population consisted of chronically infected HBV patients at the Specialized Outpatient Clinic of Viral Hepatitis of the Tropical Medicine Research Center of Rondônia—CEPEM-RO, a reference unit for the State of Rondônia and neighboring locations. All samples were collected between 2015 and 2020. Inclusion criteria were: (1) chronic hepatitis B carrier (total anti-HBc and HBsAg positive for more than 6 months); (2) detectable HBV-DNA >500 IU/mL; (3) aged > 8 and <70. Exclusion criteria were pregnancy, indigenousness, and hepatitis B patients co-infected with the hepatitis delta virus (HDV), hepatitis C virus (HCV) or human immunodeficiency virus (HIV). This study was approved by the CEPEM-RO Research Ethics Committee (Nº 3.585.613), and informed consent was obtained from all individuals who participated in the study.

### 2.2. Data Collection and Evaluation of Fibrosis Grade of Patients

Sociodemographic, clinical, laboratorial and complementary exam information were collected from medical records. Patients were classified into two groups: (i) those with mild liver disease and (ii) those with advanced liver disease, as determined by (1) a biopsy under the METAVIR classification; (2) elastography according to the degree of fibrosis; (3) fibrosis assessment scores using non-invasive methods such as the Fibrosis-4 Index (FIB-4) and AST Platelet Ratio Index (APRI) [23,24]; (4) abdominal ultrasound (EDA) images that present signs of advanced liver disease or portal hypertension such as ascites, nodular liver, and increased portal vein diameter.

Patients with at least one of the following criteria were classified as having mild liver disease: liver biopsy or elastography with a METAVIR score ≤F2, FIB-4 score <1.45, APRI score <0.5 or imaging tests with no signs of advanced liver disease. Patients with at least one of the following criteria were classified as having advanced liver disease: liver biopsy or liver elastography with a METAVIR score >F2, FIB-4 score >3.25, APRI score >0.7 or signs of advanced liver disease in imaging tests.

### 2.3. HBV Molecular Analyses

Viral DNA was extracted using 200 microliters of serum from a commercial QIAamp DNA Mini Kit (Qiagen, Hilden, Germany) following the manufacturer’s instructions. Amplification of the viral genome was performed using a nested-PCR test described previously by Barros et al., (2014) [25] which resulted in a 1306 base pair (bp) fragment corresponding to partial S/P regions of the HBV genome.

PCR products were purified using ExoSAP (Cellco, New York, NY, USA). Sequencing was performed by the Technological Platform for DNA Sequencing of the Bahia Oswaldo Cruz Foundation—FIOCRUZ/BA and at the Viral Hepatitis Laboratory of the Oswaldo Cruz Institute, using an automated Sanger sequencer ABI 3500XL (Applied Biosystems, Waltham, MA, USA). The fragments generated from the PCR reactions were sequenced using their respective forward and reverse primers. To obtain greater sequencing coverage, distinct primers were additionally used, including forward primer P781F (5′ GAR TCC CTT TWT RCC KCT RTT ACC 3′; nt781–804) and reverse primer HBV477R (5′ GGA CAV ACG GGC AAC ATA CCT T 3′; nt 477–456) [25].

### 2.4. Genotyping and Mutation Analysis

The determination of HBV genotypes was performed using phylogenetic reconstruction. Reference sequences of all HBV genotypes/subgenotypes were retrieved from the National Center of Biotechnology Information (NCBI) and comprised a final dataset of 209 sequences. The alignment was performed using the MUSCLE algorithm [26]. The phylogenetic tree was constructed using IQ-TREE v.2.2.0 [27] by the maximum likelihood method with the GTR+F+I+G4 substitution model as the best fit method measured by the ModelFinder [28]. The reliability of the phylogenetic tree was evaluated by Ultrafast Bootstrap test (1000 replicates). The final tree was edited by FigTree v.1.4.4 (http://tree.bio.ed.ac.uk/software/figtree/ (accessed on 12 January 2022)).

Sequences of each HBV isolate were analyzed using MEGA7 software [29] for prediction of clinically relevant mutations in S and P and (reverse transcriptase-RT) regions of HBV genome.

### 2.5. Molecular Modeling of HBsAg

The tridimensional structure of the S domain of HBV surface protein (HBsAg) was built by molecular modeling by I-Tasser server1 [30]. The tool implemented homology modeling of protein structure by iterative template-based fragment assembly simulations. The reference sequence HBV_RO_10 was selected for this construction. The predicted 3D structure was then processed to obtain a refined model by the use of GalaxyWEB Refine2 [31]. The Ramachandran plot was adopted as a structure validation tool and the best model was chosen for further molecular docking analysis.

### 2.6. Molecular Docking of HBsAg and Anti-HBs

The HADDOCK 2.4 server [32] was used to perform docking analysis of the HBsAg construction with antibody IgG anti-HBs (PDB id: 6VJT)3. The structure of the monoclonal antibody refers to the Fab subunit with specificity for the KPSDGN epitope, which contains some of the most frequent mutated amino acids during natural infections. We analyzed the interaction of the S domain of HBsAg-HBV_RO_10 with the antibody heavy chain. The recognition domain in HBsAg was selected by superposition with the linear epitope of the antibody as the center of the binding site with a radius of 10 Å. HADDOCK output was composed of multiple models, out of which the highest 10 clusters were selected and the top was dependent on its Z-score: the more negative the score, the better the cluster. An analysis of interactions was then performed by score data and atom interactions by PDBsum Generate tool4 [33]. Images were generated by PyMol v. 2.1.5 [34]. 

### 2.7. Statistical Analysis

The results of the descriptive analyses were represented through frequencies and measures of central tendency and dispersion. Statistical inference was adopted using Fisher’s exact test and Odds Ratio. Factors that were significant (*p* < 0.05) were included in the multivariate model (Logistic regression). All statistical analysis was performed using R v4.0.3 software [35].

## 3. Results

### 3.1. Characteristics of the Study Population

A total of 62 HBV chronically infected patients were included in the study and classified according to the severity of the liver disease. No individual was classified as cirrhotic or diagnosed with hepatocellular carcinoma. Table 1 shows the results of the clinical and laboratorial characterization of the study population.

### 3.2. Genotyping and Phylogenetic Analyses

**Figure 1:**
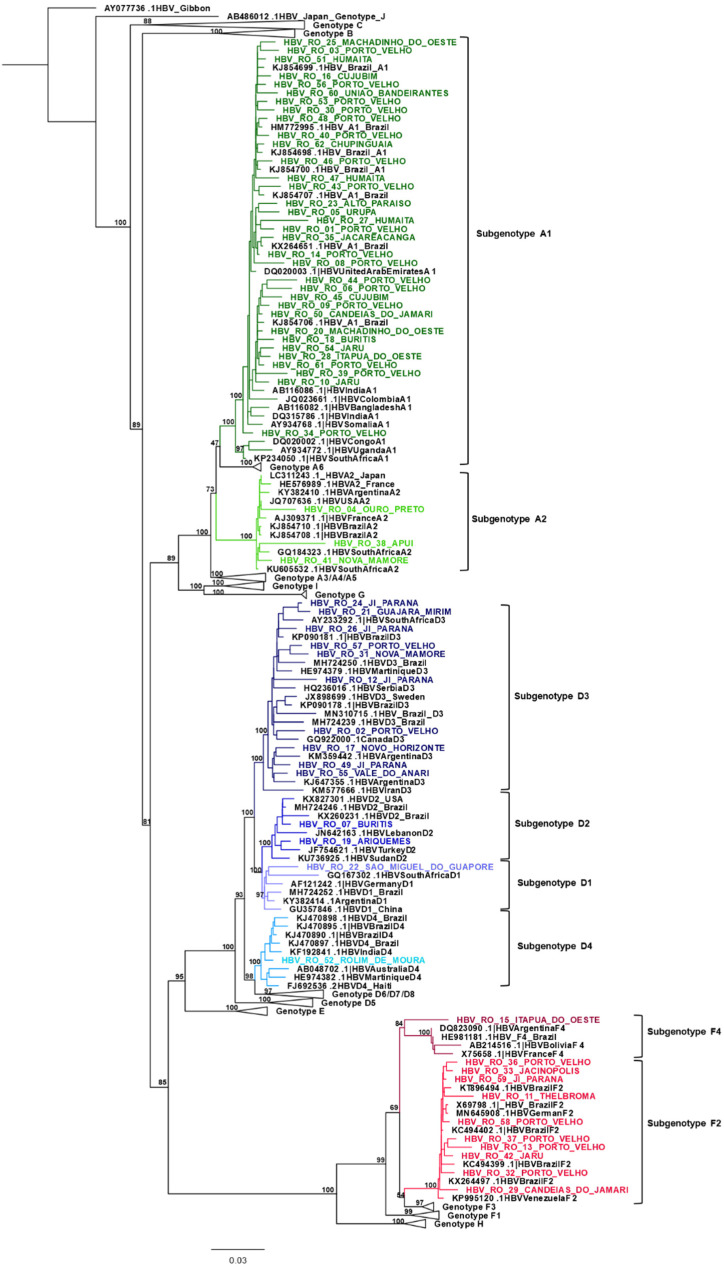
Maximum likelihood phylogenetic tree of all samples enrolled in this study (n = 62). Colours represent a genotype/subgenotype clade. Reference sequences (n = 147) are displayed in black.

Phylogenetic analyses (Figure 1) demonstrated the presence of genotypes A (59.7%), D (22.6%) and F (17.7%). Subgenotype A1 was the most prevalent, detected in 54.83% (34/62) of the samples, followed by D3 (16.13%; 10/62), F2 (16.13%; 10/62), A2 (4.84; 3/62), D2 (3.23%; 2/62), D1 (1.61%; 1/62), D4 (1.61%; 1/62) and F4 (1.61%; 1/62).

The analyses also demonstrated that subgenotypes A1, D3, F2 and D4 presented high genetic relatedness with previously published Brazilian sequences. Samples classified as genotypes A2, D1 and D2 clustered with sequences from Europe, USA, South Africa and Turkey. The sample attributed to subgenotype F4 was located in a separate branch of the phylogenetic tree.

### 3.3. Mutation Analisys and Characteristics of Samples

All samples were sequenced and analyzed for S and partial P (RT) regions. Only 58/62 had the pre-S2 region successfully sequenced. The results showed relevant deletions in the pre-S2 region in 13.79% (8/58) of the samples, mutations in the S gene in 59.68% (37/62) and in RT in 48.39% (30/62) of the subjects (Table 2). In this study, chronic hepatitis B carriers in the immuno-active or inactive phases were included. This information was provided in Table 2 for comparison with the mutations that were identified in each patient. Additional information is provided in Table S1.

### 3.4. Demographic, Clinical and Molecular Analysis of Patients with Mild and Advanced Liver Disease

Demographic, clinical and virological characteristics of individuals were evaluated according to the severity of liver disease. As shown in Table 3, most advanced liver disease patients were male, who are more likely than those with mild liver disease to present HBeAg positivity. Moreover, among all patients with the D genotype, 50% (7/14) had advanced liver disease (*p* < 0.05).

### 3.5. Molecular Modeling of HBsAg and Docking of HBsAg and Anti-HBs

**Figure 2:**
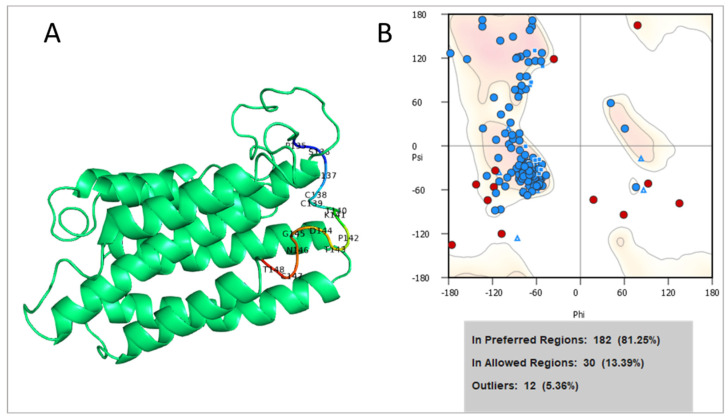
(**A**) Three-dimensional structure of HBsAg of a conserved epitope of HBV-RO-10. The antigenic region used in molecular docking is highlighted in the image; (**B**) Ramachandran plot. Blue dots and triangles represent residues in favored regions and red dots indicates the outliers residues. Images generated by PyMOL©.

The amino acid sequence of a highly conserved region of the S domain of HBsAg was submitted for homology modeling. The confidence of the model predicted by iTASSER was quantitatively measured by a C-score, where the higher value indicates stability and confidence. The best model present a C-score of −2.13. As for the refined structure, the final model was chosen by Ramachandran plot analysis. This analysis showed that 81.25% of the structure was under the favoured region; 13.39% was under the allowed region; and 5.36% was observed under the disallowed region, signalling a high quality of the predicted structure (Figure 2).

**Figure 3:**
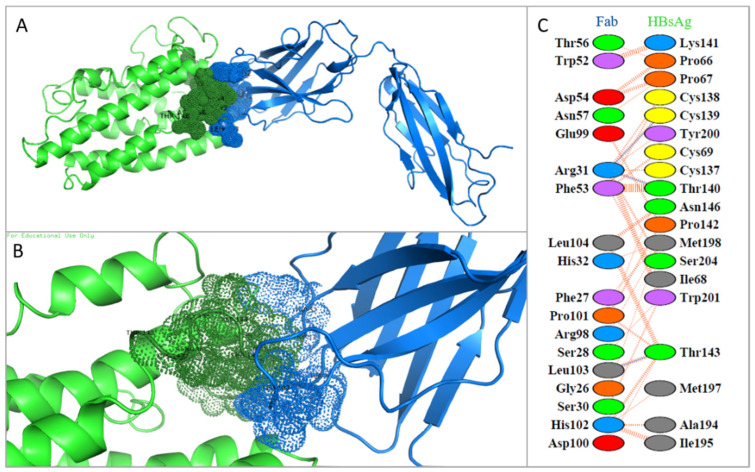
(**A**) Molecular docking between HBV-RO-10 and the heavy chain of the Fab fragment of antibody 6VJT (anti-HBs); (**B**) Site of interaction between the chains of the two proteins is highlighted; (**C**) Plot of hydrogen bonds (blue lines), nonbonded contacts (orange tick marks), and salt bridges (red lines) between residues on either side of the protein–protein interface. Images generated with PyMOL© and PDBSum©.

A docking analysis was performed to evaluate the interaction between the virus and the neutralizing antibody. Our results showed good interaction between the HBsAg construction and the antibody with a HADDOCK score value of −106.7 ± 7.1. Parameters of Van der Waals, electrostatic and desolvation energy were −69.8 +/− 5.7, −53.5 +/− 9.4 and −29.7 +/− 4.0, respectively. These results corroborated the favorable interaction during the simulation. The regions in contact are represented in Figure 3 by dots. It was observed that 18 amino acid residues of the antibody interacted with the other 19 residues of HBsAg, with emphasis on the hydrogen bonds Arg31-Cys39, Arg31-Thr140 and Leu103-Thr143. Nonbonded contacts and salt bridges also contributed to the binding. Based on the analyzed epitope, there is evidence that the study samples had higher neutralizing activity from anti-HBs antibodies that had a low probability of vaccine escape or reinfection after acute infection.

## 4. Discussion

This study provided the most comprehensive molecular characterization of HBV isolates circulating in Rondônia, Brazil, to date, including samples from the capital and rural towns. Moreover, we described important deletions in pre-S2 and clinically relevant mutations, in S, and in RT HBV genomic regions in patients with mild and advanced liver disease. 

In Brazil, the Viral Hepatitis Control Program coordinated by the Ministry of Health guides the diagnosis and antiviral treatment to chronic hepatitis B carriers, and the federal government provides free antiviral treatment to each patient who requires it. In addition, the Epidemiological Surveillance of the municipality conducts hepatitis B tests for those who came in contact with infected individuals to identify acute cases.

Advanced liver disease was significant among males and those belonging to genotype D. Progression to cirrhosis and HCC is often associated with long-term liver disease [17,18,19]. Studies have shown that those who are male, older and have decompensated cirrhosis, viral flare (presence of HBeAg or high viral loads and transaminases) and other host/viral factors are more likely to have severe liver disease in the form of advanced fibrosis [36,37,38,39,40,41]. It has been reported that genotypes A, C and F may have an increased risk of liver disease progression [42,43,44]. In this study, a significant portion of genotype D was observed among patients with advanced liver disease, providing an important finding for the general population.

Genotype circulation is in agreement with other studies performed in Brazil, in which genotype A predominates, followed by D and F [25,45,46,47,48,49,50]. Similar results were observed in previous research in the same region that described the presence of subgenotypes A1, D3, F2a, D4 and D2 [45]. Our findings showed the circulation of these subgenotypes in different proportions (A1; D3; F2; D2; D4) and reported for the first time the presence of subgenotypes A2, D1 and F4. As previously reported, A1, D3 and F2 are the most prevalent subgenotypes in the northern region [49].

Phylogenetic analysis demonstrated that samples from subgenotypes A1, D3, D4 and F2 clustered with circulating viral isolates [51,52,53,54,55,56]. On the other hand, samples from A2, D1 and D2 clustered with sequences from Europe [57], USA [58], South Africa (unpublished) and Turkey [59]. The sample assigned to subgenotype F4 was located in a separate branch of the F4 clade (Figure 1) and showed divergence 4% above the genetically closest F4 sample. As no evidence of recombination was found, complete genome analysis could provide a more accurate analysis of the genetic profile of this isolate.

As expected, subgenotype A1 sequences clustered in the Asia-American clade. Although a monophyletic origin cannot be claimed, A1-Brazilian samples presented a closer intragenetic relatedness than with sequences from Latin American, African or Asian countries. However, a further study analyzing the complete genome of a larger number of Brazilian sequences should be performed to confirm this relatedness.

In this study, 56.45% of patients presented clinically relevant mutations such as sC69STOP, Y100C, sM103I sL109M/Q, sP120T, sG130S/N, sM133T, sS/T140I, sG145R and sE164G. These are commonly associated with a reduction in affinity for and recognition of lymphocytes and neutralizing antibodies, vaccine escape, non-detection of HBsAg in diagnostic tests and occult hepatitis B [60,61,62,63,64,65]. The sG145R mutation was found in one patient, a substitution associated with vaccine escape once it promotes a crucial change in the structure of the determinant “a” epitope, thus affecting antigen recognition by neutralizing antibodies [66,67]. Nevertheless, our findings demonstrated that not all patients with an HBsAg mutation had a negative HBsAg profile, and there was no significant association with advanced liver disease progression.

Another important finding was the mutations sC69*/rtS78T, found in a 31-year-old man with advanced liver disease. The sC69* mutation results in a premature stop codon, leading to a truncated protein and significantly reducing HBsAg levels [68]. Regarding rtS78T, studies reported that this substitution enhances HBV replication with reduced susceptibility to ETV and TDF [69,70,71] despite other research demonstrating that this mutation has limited effect on drug resistance [41]. In this study, the sC69*/rtS78T carrier is under TDF therapy. This patient presented detectable HBsAg, a high viral load (6.9 UI log/mL) and high transaminase levels, thus demonstrating a poor virological response. Nevertheless, we have no evidence that this substitution occurred during the present therapy.

Pre-S2 deletions correlate strongly with progression to advanced liver disease and the development of HCC [16,72,73]. In this study, there was a high frequency (21.43%) of pre-S2 deletions among advanced liver disease patients even though it was not statistically significant.

Regarding HBV RT mutations, rtQ215H was detected in one patient with no history of antiviral therapy. This mutation has been observed in patients who received LAM or ADV therapies; nevertheless, its clinical significance for promoting drug resistance remains inconclusive [74,75]. An RtM250I mutation was observed in another patient with no history of antiviral therapy. It has been linked to ETV resistance when associated with rtL180M and rtM204V/I, however, this double-mutation was not observed in this patient [76,77]. 

RtL180M, rtS202G, rtM204V mutations were reported in a patient who received ETV therapy for 4 years until being switched to TDF. Studies have linked this triple-mutation to ETV resistance [78,79,80,81,82,83]. Although a switch from ETV to TDF therapy was adopted, this patient had advanced liver disease and did not show signs of sustained virologic response, as evidenced by a high viral load (3.98 log IU/mL) and the HBeAg+/anti-HB− phenotype.

Two patients had concurrent HBsAg/anti-HBs, a serological profile considered atypical in HBV infection. It is associated with viral mutations and host factors resulting in continuous HBV replication despite the presence of the neutralizing antibody [84,85,86]. The docking analysis showed good interaction between the HBsAg construction and HBsAg in all samples, including those with the HBsAg/anti-HBs positive phenotype. These data confirm that all samples enrolled in this study had high neutralizing activity from anti-HBs neutralizing antibodies, suggesting that the concurrent HBsAg/anti-HBs may reflect the existence of a secondary viral subpopulation. In addition, our results showed no significant mutations that could explain these two cases, thus requiring further subpopulation investigation.

## 5. Conclusions

This study provided new data on HBV genetic surveillance and clinical outcomes and critical information for disease control. We assessed the genetic variability of HBV in Western Amazonia and found varied genotypic distribution in different locations as well as significant mutations related to immune escape and drug-resistance.

## Figures and Tables

**Table 1:** Demographic, laboratory and clinical characteristics of patients.

Feature	Value
**Demographic features**	
Male/Female	43/29
Age (years, Mean ± SD)	42.17 ± 10.41
**Laboratory features**	
HBsAg positive (n, %)	62 (100%)
Anti-HBs positive (n, %)	2 (3.22%)
HBeAg positive (n, %)	6 (9.67%)
Anti-HBe positive (n, %)	5 (8.06%)
ALT (U/L, Mean ± SD) *	37.73 ± 49.68
AST (U/L, Mean ± SD) *	35.69 ± 42.61
Viral load (Log 10 UI/mL, Mean ± SD)	4.13 ± 1.26
**Liver disease features**	
Advanced liver disease (n, %)	17 (27.42%)
Mild liver disease (n, %)	45 (72.58%)

* One patient had no ALT and AST values; Abbreviations: SD—Standard Deviation.

**Table 2:** Characteristics of samples with clinically relevant mutations.

Sample	Liver Disease	Treatment	CHB Phase	Genotype	RT Mutations	Pre-S/S Mutations
HBV_RO_01	Mild	No	Inactive	A1	-	-
HBV_RO_02	Mild	No	Inactive	D3	rtN76D, rtQ215H *	sQ30M, sP203R
HBV_RO_03	Mild	No	Inactive	A1		sV14G, sG130S *, sL216STOP *
HBV_RO_04	Advanced	No	Inactive	A2	rtN/T118D, rtI187L, rtV190M	sV14G, sS34L, sW74L, sF85Y, sP120T *, sV190A, sY200F
HBV_RO_05	Advanced	TDF for 3 months	Immune-Active	A1	-	sL42R, sM133T *, S/T140I *, sV184A
HBV_RO_06	Mild	No	Inactive	A1	rtG25R, rtR110G, rtL229V, rtC314S	sQ16H
HBV_RO_07	Mild	No	Inactive	D2		pre-S2Δ1 *
HBV_RO_08	Mild	No	Inactive	A1	stS117Y, rtN/T118D	pre-S2Δ8-23 *, sF85C, sL109M *, sL209V, sS210K
HBV_RO_09	Mild	No	Inactive	A1	rtQ288L	-
HBV_RO_10	Mild	No	Inactive	A1	rtN/Q139E	sY100C *
HBV_RO_11	Advanced	No	Immune-Active	F2	rtM250I *, rtI265M	sI208T
HBV_RO_12	Advanced	TDF for 2 months	Inactive	D3	rtN76D, rtW284C, rtC287D, rtrtI290S	-
HBV_RO_13	Advanced	No	Inactive	F2	-	pre-S2Δ13-22 *, sV14G, sT23I, sG130S *, sF170S
HBV_RO_14	Advanced	No	Inactive	A1	-	sG130S *
HBV_RO_15	Mild	No	Inactive	F4	rtH35Q, rtN279H	sF/L8H, sF20S, sL26R, sT27K
HBV_RO_16	Mild	No	Inactive	A1	-	sG10R, sC76Y, sR78Q
HBV_RO_17	Advanced	No	Immune-Active	D3	-	pre-S2Δ18-22 *, sT189I
HBV_RO_18	Mild	No	Inactive	A1	-	pre-S2Δ5-8 *, sL91H, sG130N *
HBV_RO_19	Advanced	No	Immune-Active	D2	-	sC76Y
HBV_RO_20	Mild	No	Inactive	A1	-	sE164G *
HBV_RO_21	Advanced	No	Immune-Active	D3	-	-
HBV_RO_22	Mild	No	Inactive	D1	rtV27I, rtM309K	-
HBV_RO_23	Mild	No	Inactive	A1	rtR110G	-
HBV_RO_24	Mild	No	Inactive	D3	-	-
HBV_RO_25	Advanced	No	Inactive	A1	-	-
HBV_RO_26	Mild	No	Inactive	D3	-	-
HBV_RO_27	Mild	No	Inactive	A1	rtR110G, rtQ215H *	sG10R, sM103I *
HBV_RO_28	Mild	No	Inactive	A1	-	-
HBV_RO_29	Mild	No	Inactive	F2	rtS213T	-
HBV_RO_30	Advanced	No	Immune-Active	A1	rtL229M	pre-S2Δ20-22 *
HBV_RO_31	Advanced	No	Inactive	D3	-	-
HBV_RO_32	Mild	No	Inactive	F2	-	-
HBV_RO_33	Mild	No	Inactive	F2	rtH35Q	sL9Q, sL21W, sL26R, sT27K
HBV_RO_34	Mild	No	Inactive	A1	-	-
HBV_RO_35	Mild	No	Inactive	A1	rtV142D, rtV214I	sI3V
HBV_RO_36	Mild	No	Inactive	F2	rtH35Q	sL26R, sT27K
HBV_RO_37	Mild	No	Inactive	F2		sV14G
HBV_RO_38	Mild	No	Inactive	A2	rtL217R, rtP281H, rtM309K	sL13Q, sR79L, sL209V, sS210R, sP214L
HBV_RO_39	Mild	No	Inactive	A1	-	sG7K, sG145R *, sV180A, sP214L
HBV_RO_40	Mild	No	Inactive	A1	-	-
HBV_RO_41	Mild	No	Inactive	A2	rtN76K, rtL217R	pre-S2Δ13-22 *, sL21S, sR79H, sL209V
HBV_RO_42	Mild	No	Inactive	F2	rtH94Y	-
HBV_RO_43	Mild	No	Inactive	A1	rtR110G	-
HBV_RO_44	Mild	No	Inactive	A1	rtS57P, rtC314S	sL21S, sP29L, sS34L, sI208T
HBV_RO_45	Advanced	ETV for 5 years; TDF for 1 year (after ETV)	Immune-Active	A1	rtL180M *, rtS202G *, rtM204V *	sI195M
HBV_RO_46	Mild	No	Inactive	A1	-	-
HBV_RO_47	Mild	No	Inactive	A1	rtA87E, rtR110G, rtY111D, rtG127E	sT23I, sP70H, sG119R *
HBV_RO_48	Mild	No	Inactive	A1	rtR110G	sL77R, sL109Q *
HBV_RO_49	Advanced	TDF for 4 years	Immune-Active	D3	rtN76D, rtS78T *	sC69STOP *
HBV_RO_50	Mild	No	Immune-Active	A1	-	sM133T *
HBV_RO_51	Mild	No	Inactive	A1	rtS230Y	-
HBV_RO_52	Advanced	No	Immune-Active	D4	-	-
HBV_RO_53	Advanced	No	Immune-Active	A1	-	sM133T *
HBV_RO_54	Mild	No	Inactive	A1	rtG104C	-
HBV_RO_55	Advanced	ETV for 2 months	Inactive	D3	rtG104C	sP120T *
HBV_RO_56	Mild	No	Inactive	A1	rtR110G	-
HBV_RO_57	Mild	No	Immune-Active	D3	-	-
HBV_RO_58	Mild	No	Inactive	F2	-	-
HBV_RO_59	Mild	No	Immune-Active	F2	-	-
HBV_RO_60	Mild	No	Inactive	A1	-	pre-S2Δ13-14 and Δ18-22 *, sV180A, sP203Q
HBV_RO_61	Mild	No	Inactive	A1	-	-
HBV_RO_62	Mild	No	Inactive	A1	-	-

* Clinical relevance mutations in RT and pre-S/S gene are marked in bold.

**Table 3:** Frequency of variables according to liver disease classification.

Variable	Liver Disease		ORc (CI 95%)	*p*	ORad (CI 95%)	*p*
	Mild (n = 45)	Advanced (n = 17)				
Age						
<30 (n, %)	6 (13.3)	1 (5.9)		-		
31–40 (n, %)	18 (40.0)	5 (29.4)	1.67 (0.16–17.25)	0.668	-	-
41–50 (n, %)	9 (20.0)	8 (47.1)	5.33 (0.52–54.33)	0.157	-	-
>50 (n, %)	12 (26.7)	3 (17.6)	1.50 (0.13–17.67)	0.747	-	-
Gender						
Female (n, %)	26 (47.8)	3 (17.64)	**6.20 (1.45–38.32)**	**0.009**	**9.57 (1.46–62.86)**	**0.019**
**Male (n, %)**	**19 (42.2)**	**14 (82.35)**				
HBeAg						
Negative (n, %)	43 (95.56)	13 (76.47)	6.37 (0.81–77.81)	0.043	3.63 (0.32–41.45)	0.299
Positive (n, %)	2 (4.44)	4 (23.53)				
ALT status *						
Normal	41 (93.18)	9 (52.94)	11.47 (2.23–80.65)	<0.001	3.63 (0.46–28.84)	0.222
Increased	3 (6.82)	8 (47.06)				
Viral Load (UI/mL)						
≤20,000 (n, %)	36 (80.00)	8 (47.06)	4.37 (1.15–17.61)	0.025	1.56 (0.21–11.73	0.667
>20,000 (n, %)	9 (20.00)	9 (52.94)				
Genotype						
A (n, %)	29 (64.44)	8 (47.05)		-		
**D (n, %)**	**7 (15.56)**	**7 (41.18)**	**5.71 (1.50–21.84)**	**0.011**	**7.44 (1.19–46.64)**	**0.032**
F (n, %)	9 (20)	2 (11.77)	0.95 (0.17–5.42)	0.956	-	-
Pre-S2 deletions **						
No (n, %)	39 (88.64)	11 (78.57)	2.09 (0.28–12.89)	0.385	-	-
Yes (n, %)	5 (11.36)	3 (21.43)				
HBsAg mutations						
No (n, %)	23 (51.1)	6 (35.3)	1.90 (0.53–7.40)	0.392	-	-
Yes (n, %)	22 (48.9)	11 (64.7)				

* One patient had no ALT values; ** Only 58 samples were included in this analysis. Abbreviations: ORc—Crude Odds Ratio; ORad—Adjusted Odds Ratio; CI—Confidence Interval. Statistical significance appeared in bold.

## Data Availability

Sequences submitted in this study are available at NCBI GenBank with the accession numbers: OM181481-OM181514 and ON529684-ON529711.

## Supplementary Materials

The following supporting information can be downloaded at: https://www.mdpi.com/article/10.3390/v14102100/s1. Table S1: Additional data from the study participants.
