# Supplementary material for: Genomic Variability of Hepatitis B Virus Circulating in Brazilian Western Amazon
Source: Viruses. 2022 Sep 22;14(10):2100. doi: 10.3390/v14102100 (PMC9611064; doi:10.3390/v14102100)
Supplement: Supplementary file 1 [file viruses-14-02100-s001.zip › viruses-1839733-supplementary.pdf]

**Table S1.** Additional data from the study participants.

| Sample | Age | Sex | Location | HBsAg/anti-HBs | HBeAg/anti-HBe | Viral load (Log10 UI/ml) | ALT (U/L) | AST (U/L) |
|---|---|---|---|---|---|---|---|---|
| HBV_RO_01 | 50 | Female | Porto Velho - RO | +/- | -/+ | 3,32 | 16 | 22 |
| HBV_RO_02 | 38 | Female | Porto Velho - RO | +/- | -/+ | 3 | 26 | 18 |
| HBV_RO_03 | 52 | Female | Porto Velho - RO | +/- | -/+ | 2,91 | 16 | 24 |
| HBV_RO_04 | 58 | Male | Ouro Preto - RO | +/- | -/+ | 3,1 | 22 | 34 |
| HBV_RO_05 | 47 | Male | Urupá - RO | +/- | -/- | 7,64 | 97,3 | 59,9 |
| HBV_RO_06 | 58 | Female | Porto Velho - RO | +/- | -/+ | 4,58 | 16 | 23 |
| HBV_RO_07 | 33 | Male | Buritus - RO | +/- | -/+ | 3,31 | 31 | 32 |
| HBV_RO_08 | 53 | Male | Porto Velho - RO | +/- | -/+ | 3,2 | 30 | 28 |
| HBV_RO_09 | 39 | Female | Porto Velho - RO | +/- | -/+ | 3,46 | 16 | 15 |
| HBV_RO_10 | 43 | Male | Jarú - RO | +/- | -/+ | 3,66 | 20 | 24 |
| HBV_RO_11 | 35 | Male | Theobroma - RO | +/- | +/+ | 5,77 | 65 | 54 |
| HBV_RO_12 | 33 | Male | Ji Paraná - RO | +/- | -/+ | 3,99 | 30,7 | 25,8 |
| HBV_RO_13 | 53 | Male | Porto Velho - RO | +/- | -/+ | 2,58 | 32 | 28 |
| HBV_RO_14 | 29 | Male | Porto Velho - RO | +/- | -/+ | 3,88 | 23 | 43,7 |
| HBV_RO_15 | 34 | Male | Itapoã D'Oeste - RO | +/- | -/+ | 4,76 | 17,7 | 22,7 |
| HBV_RO_16 | 41 | Female | Cujubim - RO | +/- | -/+ | 4,25 | 34 | 26 |
| HBV_RO_17 | 44 | Male | Novo Horizonte - RO | +/- | +/+ | 7,43 | 35,9 | 66,3 |
| HBV_RO_18 | 41 | Female | Buritis - RO | +/- | -/+ | 2,76 | 9 | 15 |
| HBV_RO_19 | 46 | Female | Ariquemes - RO | +/- | -/+ | 4,58 | 347 | 219 |
| HBV_RO_20 | 35 | Female | Machadinho D'Oeste - RO | +/- | -/+ | 5,51 | 14 | 17 |
| HBV_RO_21 | 40 | Male | Guajará Mirim - RO | +/- | -/+ | 6,21 | 72 | 63 |
| HBV_RO_22 | 58 | Female | São Miguel do Guaporé - RO | +/- | -/+ | 5,34 | 15 | 18 |
| HBV_RO_23 | 37 | Male | Alto Paraíso - RO | +/- | -/+ | 3,62 | 17 | 48 |
| HBV_RO_24 | 29 | Female | Ji Paraná - RO | +/- | -/+ | 3,03 | 42 | 32 |
| HBV_RO_25 | 50 | Male | Machadinho D'Oeste - RO | +/- | -/+ | 2,88 | 39 | 30 |
| HBV_RO_26 | 29 | Female | Ji Paraná - RO | +/- | -/+ | 4,25 | 18 | 23 |
| HBV_RO_27 | 56 | Male | Humaitá - AM | +/- | -/+ | 2,84 | 15 | 20 |
| HBV_RO_28 | 38 | Male | Itapuã D'Oeste - RO | +/- | -/+ | 3,39 | 39 | 26 |
| HBV_RO_29 | 40 | Male | Candeias do Jamari - RO | +/- | -/+ | 2,54 | 19,23 | 22,62 |
| HBV_RO_30 | 54 | Male | Porto Velho - RO | +/- | -/+ | 5,5 | 48 | 44 |
| HBV_RO_31 | 41 | Male | Nova Mamoré - RO | +/- | -/+ | 3,49 | 18 | 19 |
| HBV_RO_32 | 34 | Male | Porto Velho - RO | +/- | -/+ | 4,01 | 23 | 27 |
| HBV_RO_33 | 37 | Male | Jacinópoles - RO | +/- | -/+ | 3,1 | 22 | 29 |
| HBV_RO_34 | 30 | Female | Porto Velho - RO | +/- | -/+ | 3,55 | 9 | 20 |
| HBV_RO_35 | 59 | Female | Jacareacanga - PA | +/- | -/+ | 2,57 | 17 | 22 |
| HBV_RO_36 | 54 | Female | Porto Velho - RO | +/- | -/+ | 3,32 | 19 | 21 |
| HBV_RO_37 | 54 | Female | Porto Velho - RO | +/- | -/+ | 3,46 | 14 | 18 |
| HBV_RO_38 | 47 | Female | Apuí - AM | +/- | -/+ | 3,09 | 20 | 20 |

| | | | | | | | | |
|---|---|---|---|---|---|---|---|---|
| HBV_RO_39 | 63 | Female | Porto Velho - RO | +/- | -/+ | 4,27 | 25 | 23 |
| HBV_RO_40 | 27 | Male | Porto Velho - RO | +/- | -/+ | 3,82 | 34 | 32 |
| HBV_RO_41 | 27 | Male | Nova Mamoré - RO | +/- | -/+ | 3,45 | 34 | 35 |
| HBV_RO_42 | 40 | Male | Jarú - RO | +/- | -/+ | 3,94 | 38 | 23 |
| HBV_RO_43 | 61 | Female | Porto Velho - RO | +/- | -/+ | 3,71 | 21 | 26,3 |
| HBV_RO_44 | 51 | Male | Porto Velho - RO | +/- | -/+ | 3,08 | 18 | 19 |
| HBV_RO_45 | 43 | Male | Cujubim - RO | +/- | +/- | 3,98 | 55 | 46 |
| HBV_RO_46 | 32 | Female | Porto Velho - RO | +/- | -/+ | 3,56 | 25 | 20 |
| HBV_RO_47 | 25 | Female | Humaitá - AM | +/- | -/+ | 4,42 | 11 | 17 |
| HBV_RO_48 | 66 | Female | Porto Velho - RO | +/+ | -/+ | 3,88 | 23 | 25 |
| HBV_RO_49 | 31 | Male | Jí Paraná - RO | +/- | -/- | 6,9 | 179,6 | 83,6 |
| HBV_RO_50 | 45 | Male | Candeias do Jamari - RO | +/- | +/- | 6,64 | 73 | 31 |
| HBV_RO_51 | 42 | Male | Humaitá - AM | +/- | -/+ | 3,45 | 28 | 26 |
| HBV_RO_52 | 45 | Female | Rolim de Moura - RO | +/- | +/- | 6,34 | 21 | 24 |
| HBV_RO_53 | 37 | Male | Porto Velho - RO | +/- | -/+ | 5,4 | 132 | 283 |
| HBV_RO_54 | 34 | Female | Jarú - RO | +/+ | -/+ | 5,72 | 21 | 24 |
| HBV_RO_55 | 49 | Female | Vale do Anari - RO | +/- | -/+ | 3,62 | 19,1 | 18,9 |
| HBV_RO_56 | 45 | Female | Porto Velho - RO | +/- | -/+ | 3,31 | 16 | 16 |
| HBV_RO_57 | 32 | Male | Porto Velho - RO | +/- | -/+ | 4,91 | 75 | 22 |
| HBV_RO_58 | 22 | Female | Porto Velho - RO | +/- | -/+ | 4,12 | 17 | 18 |
| HBV_RO_59 | 33 | Female | Jí Paraná - RO | +/- | +/+ | 6,63 | 32,3 | 21 |
| HBV_RO_60 | 44 | Female | União Bandeirantes - RO | +/- | -/+ | 4,23 | - | - |
| HBV_RO_61 | 35 | Male | Porto Velho - RO | +/- | -/+ | 2,87 | 21 | 22 |
| HBV_RO_62 | 37 | Male | Chupinguaia - RO | +/- | -/+ | 3,67 | 18 | 21 |
